# Acalculous Cholecystitis From Kawasaki Disease in a Three-Month-Old Girl: A Rare Sign at an Uncommon Age

**DOI:** 10.7759/cureus.64929

**Published:** 2024-07-19

**Authors:** Hansa Sriphongphankul, Jirayut Jarutach, Thampapon Chaisujyakorn, Supika Kritsaneepaiboon, Phurich Janjindamai

**Affiliations:** 1 Department of Pediatrics, Prince of Songkla University, Songkhla, THA; 2 Department of Radiology, Prince of Songkla University, Songkhla, THA

**Keywords:** non-infectious cholecystitis, incomplete kawasaki disease, infant, atypical kawasaki disease, acute acalculous cholecystitis

## Abstract

We report a case of a previously healthy three-month-old girl who presented with acute fever, watery diarrhea, and right upper abdominal guarding. Abdominal ultrasonography findings were compatible with acute acalculous cholecystitis. Initially, antibiotics were administered for a total of eight days without improvement. Hence, atypical Kawasaki disease (KD) was suspected despite the absence of classical disease manifestations and her uncommon age. The diagnosis was made using alternative diagnostic criteria and echocardiography. After KD was diagnosed, high-dose intravenous immunoglobulin G and aspirin were administered on day 9 of disease onset. Her clinical condition significantly improved within 24 hours, and she recovered well without complications during the 1.5 years of follow-up.

## Introduction

Acute acalculous cholecystitis (AAC) constitutes not only an uncommon gallbladder disease in healthy children but also a rare clinical presentation of Kawasaki disease (KD). However, AAC resulting from KD exhibits a higher prevalence in infancy and early childhood than in older age. Atypical KD in infants is frequently challenging to diagnose. Delayed diagnosis and treatment of KD may result in several complications, including coronary artery aneurysm, necessitating prolonged medication and potentially impairing the quality of life [1]. We report a case of a three-month-old girl who presented with KD at an atypical age.

## Case presentation

A three-month-old previously healthy girl was admitted due to high-grade fever with acute watery diarrhea for one day. She was the second child of a mother with well-controlled gestational diabetes, born at 39 weeks of gestation via cesarean section (C/S) due to a history of C/S. The maternal history during pregnancy was otherwise unremarkable. The child has been exclusively breastfed and was fully immunized up to two months of age. There was no history of fever, infection, diarrhea, or jaundice among family members in the three months following the patient's birth. Physical examination revealed a body temperature of 39°C, a pulse rate of 160/min, dry lips, and hyperactive bowel sounds without abdominal tenderness. The remainder of the physical examination was within normal limits. Her initial complete blood count (CBC) showed white blood cells (WBC) of 22,870/mm³, neutrophils (N) of 68%, lymphocytes (L) of 25%, hematocrit (Hct) of 36.1%, and platelets (Plt) of 313,000/mm³. Stool examination exhibited neither white nor red blood cells. She was provisionally diagnosed with acute gastroenteritis and mild dehydration. Because occult bacteremia remained a concern, ceftriaxone was administered.

**Figure 1:**
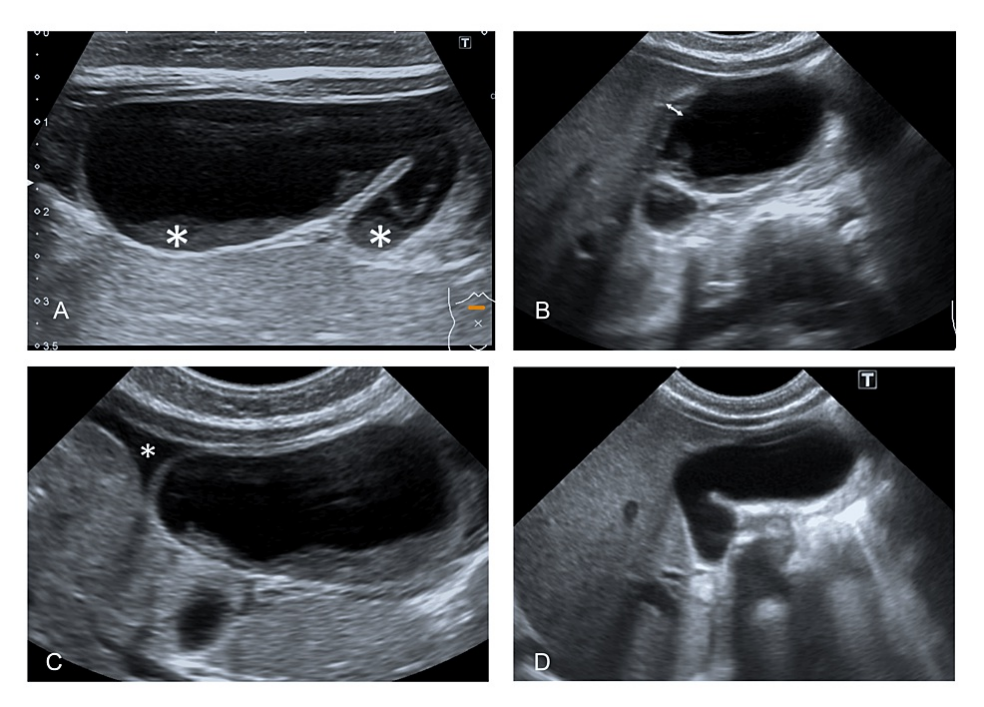
Hepatobiliary ultrasound A) Marked dilatation of the gallbladder with internal bile sludge; B) Gallbladder wall thickening; C) Pericholecystic fluid; D) Resolution of the previously detected distended gallbladder and internal bile sludge without pericholecystic fluid.

On hospital day (HD) 3, she developed a sudden onset of intermittent crying, refusal to eat, a high-grade fever, abdominal distension, generalized tenderness, and guarding. The abdominal radiograph revealed soft tissue density at the subhepatic region and inferiorly displaced bowel loops. The hepatobiliary ultrasound showed marked dilatation of the gallbladder with internal bile sludge, approximately 4.3 x 1.7 cm. Gallbladder wall thickening and pericholecystic fluid were also observed (Figure 1).

AAC was considered. Due to clinical deterioration, a bacterial infection was suspected, leading to a switch in antibiotics to meropenem for drug-resistant bacteria, such as *Salmonella* spp. or *Escherichia coli* (*E. coli*). Despite three days of treatment, fever and diarrhea persisted, with only slight improvements in appetite and abdominal guarding. Stool tests showed mucous, moderate WBC, and no occult blood. Ciprofloxacin was added on HD 6 to target drug-resistant *Salmonella*.

**Figure 2:**
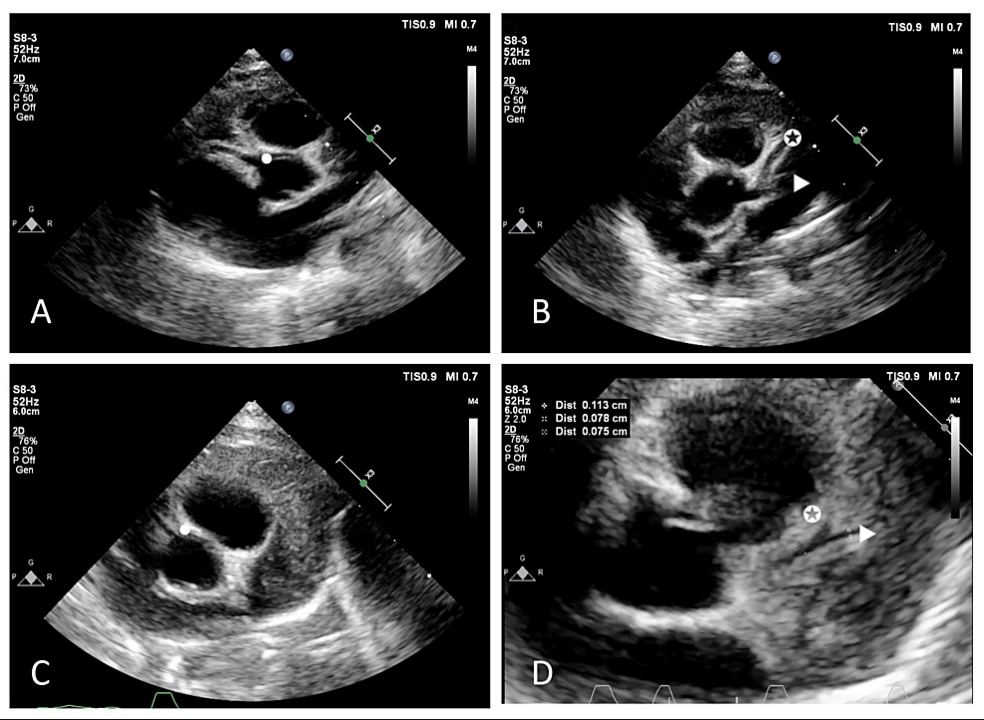
Echocardiogram (modified high parasternal short-axis view at the level of the aortic valve) A) and B) Presence of perivascular brightness with generalized dilatation and small aneurysm of RCA (dot), LAD (star), and LCx (triangle). C) and D) Follow-up echocardiogram showing regressed to normal size of all coronary arteries. LAD, Left anterior descending coronary artery; LCx, Left circumflex artery; RCA, Right coronary artery

Despite ongoing symptoms such as fever, diarrhea, and abdominal guarding, and with no bacterial growth in blood and rectal cultures on HD 1, 3, and 6, antibiotics were discontinued on HD 8. A blood test using real-time polymerase chain reaction (PCR) for human herpesviruses (HHVs) 1-6, including Epstein-Barr virus and cytomegalovirus, reported a negative result. KD was considered a potential non-infectious cause for her symptoms. Given the absence of KD's typical signs, except for a fever lasting more than five days, additional tests conducted on HD 8 showed an elevation in erythrocyte sedimentation rate and C-reactive protein (CRP) of 79 mm/h and 178 mg/dL, respectively. CBC revealed WBC of 34,840 cells/mm³ (N 77%, L 8%), Hct of 25.5%, and Plt of 929,000/mm³. The liver function test showed normal liver enzymes with albumin of 2.9 g/dL. Urinary analysis was normal. Echocardiography demonstrated perivascular brightness and generalized dilatation of all coronary arteries with a small aneurysm (z-score of 4.0-4.5) (Figure 2).

Atypical KD was diagnosed on HD 9. A single high dose of intravenous immunoglobulin G (IVIG), 2 g/kg, and high-dose aspirin (90 mg/kg/day) were administered. Within 24 hours after IVIG and aspirin administration, fever and abdominal guarding receded. The frequency of defecation decreased and returned to normal by HD 14. Laboratory tests before discharge revealed WBC of 13,880 cells/mm³, Hct of 30%, Plt of 1,188,000/mm³, CRP of 5.2 mg/dL, and albumin of 3.5 g/dL. Follow-up abdominal USG revealed the resolution of the previously detected distended gallbladder and internal bile sludge without pericholecystic fluid (Figure 2D). She was administered low-dose aspirin (5 mg/kg/day) and a ferrous sulfate supplement at home and was advised to avoid live vaccines for at least 11 months after IVIG administration.

An echocardiogram on day 25 after IVIG administration revealed a normal coronary artery (Figure 2D). The follow-up blood tests are shown in Table 1.

**Table 1:** The laboratory findings before and after treatment with IVIG From day -9 (admission) to day 90, relative to the diagnosis date of atypical Kawasaki disease (KD), showing sequential days before and after diagnosis for monitoring laboratory findings IVIG, Intravenous immunoglobulin G; AAC, Acute acalculous cholecystitis; KD, Kawasaki disease; WBC, White blood cell; Hb, Hemoglobin; Hct, Hematocrit; RBC, Red blood cell; MCV, Mean corpuscular volume; MCH, Mean corpuscular hemoglobin; MCHC, Mean corpuscular hemoglobin concentration; RDW, Red blood cell distribution width; BUN, Blood urea nitrogen; ESR, Erythrocyte sedimentation rate; CRP, C-reactive protein; TB, Total bilirubin; DB, Direct bilirubin; AST, Aspartate aminotransferase; ALT, Alanine aminotransferase; ALP, Alkaline phosphatase; Alb, Albumin

Laboratory finding	Day -9 (admission date)	Day -6 (AAC was diagnosed)	Day -3	Day -1 (atypical KD was suspected)	Day 0 (atypical KD was diagnosed, and IVIG was given)	Day 5 (discharge date)	Day 10	Day 25	Day 90
WBC (x10^3^/mm^3^)	22.87	10.10	23.96	34.84	-	13.88	14.96	10.69	9.4
Neutrophil (%)	68	58	66	77	-	29	30	28	23
Lymphocyte (%)	25	22	23	8	-	61	61	58	65
Band (%)	-	11	1	-	-	-	-	-	-
Monocyte (%)	6	1	8	9	-	8	4	6	3
Hb (g/dL)	11.5	11.2	9.5	7.7	-	9.4	9.8	9.3	11.7
Hct (%)	36.1	34.5	29.9	25.5	-	30.1	30.7	29.8	36.4
RBC (x10^6^/mm^3^)	4.05	4.01	3.48	2.83	-	3.51	3.7	3.63	4.51
MCV (fL)	89.1	86	85.9	90.1	-	85.8	83.2	82.1	80.7
MCH (pg)	28.4	27.9	27.3	27.2	-	26.8	26.6	25.6	25.9
MCHC (g/dL)	31.9	32.5	31.8	30.2	-	31.2	31.9	31.2	32.1
RDW (%)	12.2	12.3	13.2	13.7	-	13.8	13.9	13.4	13.5
Platelet (x10^3^/mm^3^)	313	179	612	929	-	1188	915	340	402
BUN (mg%)	4	2	4.4	5	-	6.6	-	-	-
Creatinine (mg%)	0.3	0.25	0.2	0.2	-	0.2	-	-	-
Sodium (mmol/L)	135	136	137	134	-	135	-	-	-
Potassium (mmol/L)	3.9	3.9	4.5	4.4	-	4.5	-	-	-
Chloride (mmol/L)	101.1	105.2	102.9	100.2	-	101.5	-	-	-
Bicarbonate (mmol/L)	16	20	23	22	-	19	-	-	-
ESR (mm/h)	-	-	-	79	-	71	23	-	-
CRP (mg/dL)	-	-	-	178.38	-	5.21	0.93	-	-
TB (mg/dL)	-	1.69	0.7	0.54	-	0.46	0.51	-	-
DB (mg/dL)	-	1.48	0.29	0.31	-	0.24	0.25	-	-
AST (U/L)	-	43	18	21	-	38	34	-	-
ALT (U/L)	-	43	18	10	-	17	22	-	-
ALP (U/L)	-	93	92	96	-	141	178	-	-
Alb (g/dL)	-	3.2	3	2.9	-	3.5	4.1	-	-

The continuation of a low dose of aspirin was suggested until the age of 11 months. During the 1.5-year follow-up period, she continued to recover well without complications.

## Discussion

AAC, an inflammation of the gallbladder in the absence of gallstones, is uncommon in children. It represents 50-70% of all cases of acute cholecystitis in children [2]. The clinical manifestations include fever (100%), anorexia (78%), jaundice (40%), abdominal pain (25%), vomiting (32%), and diarrhea (25%) [3]. The predisposing causes of AAC in previously healthy children are predominantly infectious (50-70%), including viral infections (such as Epstein-Barr virus, cytomegalovirus, and hepatitis A) and bacterial infections (such as *Mycoplasma* spp., *Salmonella* spp., *Streptococcus* spp., and *E. coli*) [2-4]. Systemic non-infectious diseases account for 14-26% of cases and include conditions such as KD and systemic lupus erythematosus [3,4]. Interestingly, systemic non-infectious diseases are more prevalent in infants and young children than in older individuals and are mostly caused by KD [4]. Yi et al. have reported the prevalence of predisposing causes in AAC among children under two years to be systemic non-infectious diseases (primarily KD) and systemic infections at 31.7% and 26.8%, respectively [4].

KD is an acute, systemic vasculitis of medium-sized vessels that mostly affects children under five years of age. The typical age at presentation is six months to two years old [5]. The prevalence of KD in children under five years is higher in Asian countries such as Japan, Korea, and Taiwan and has been reported to be 264, 134, and 66 per 100,000 children, respectively. The prevalence is lower in non-Asian countries such as the United Kingdom and Australia and has been reported to be 8.39 and 9.34 per 100,000 children, respectively [1]. The diagnosis of KD is based on the American Heart Association diagnostic criteria for classic KD, including persisting fever for more than five days with at least four of the following five features: bilateral nonpurulent conjunctivitis, rash, cervical lymphadenopathy, and mucocutaneous changes. Atypical (incomplete) KD should be considered in children with unexplained fever for greater than or equal to five days in addition to the presence of two to three principal clinical features of KD [5]. Treatment with IVIG and high-dose aspirin within 10 days of illness decreases the risk of coronary aneurysms from 25% to less than 5% [1].

We found scant literature on the prevalence and clinical presentation of patients with KD younger than one-year-old. The proportion of patients with KD aged less than or equal to six months and three months, in relation to all patients, is low - approximately 10% and 2.2%, respectively, in Korea, similar to 11.2% and 1.7% in Japan [6]. In America, the proportion of patients with KD younger than one year and less than or equal to six months was 13% and 3%, respectively [7]. However, the prevalence of atypical KD is more common in children under one-year-old than in older ones, especially those less than or equal to three months old (35-43%) [8,9]. All reported cases exhibited at least one classical manifestation of KD. Minich et al. found an age of under six months and atypical KD to be predictors of diagnosis after day 10 [10]. Moreover, some studies have reported that infants under six months old are associated with more cardiological complications, such as coronary artery aneurysm or dilatation, giant coronary artery aneurysm, pericardial effusion, or tricuspid or mitral valve regurgitation [7-9,11].

Gastrointestinal manifestations in KD, including gallbladder hydrops, AAC, bile duct inflammation, hepatitis, paralytic ileus, appendicular vasculitis, hemorrhagic duodenitis, and pancreatitis, also occur rarely; however, no clear prevalence has been reported [12-16]. Yi et al. found AAC prevalence to be 36% (24/67) in patients with KD who developed hepatobiliary manifestations, with 58% (14/24) of AAC patients having abnormal coronary arteries and 65.2% (15/24) being IVIG responsive [15]. Recently, Lipe and Bridges, and Sejeeni et al. have reported cases of atypical KD with AAC [17,18]. The review of the previous reports on KD with AAC is summarized in Table 2.

**Table 2:** Review of atypical KD cases associated with AAC KD, Kawasaki disease; AAC, Acute acalculous cholecystitis; RUQ, Right upper quadrant; RLQ, Right lower quadrant; WBC, White blood cell; ESR, Erythrocyte sedimentation rate; CRP, C-reactive protein; TB, Total bilirubin; DB, Direct bilirubin; ALP, Alkaline phosphatase; IVIG, Intravenous immunoglobulin G; Hb, Hemoglobin; AST, Aspartate aminotransferase; ALT, Alanine aminotransferase

Author	Age (gender)	Clinical manifestation		Supplemental laboratory criteria result for atypical KD	Hepatobiliary ultrasound finding	Echocardiogram finding	Treatment and outcome
		KD	AAC				
Lipe and Bridges (2019) [17]	8 (boy)	Fever, injected conjunctiva, desquamation of the lips	Diarrhea, vomiting, RUQ, RLQ pain and guarding	Pyuria	A distended gallbladder with scant pericholecystic fluid	No evidence of coronary artery aneurysms	High-dose aspirin, IVIG: Recovery
				WBC: 13,200/mm^3^			
				ESR: 28 mm/h			
				CRP: 6.2 mg/dL			
				TB: 3.6, DB: 2.8 mg/dL, ALP: 428 U/L			
Sejeeni et al. (2023) [18]	5.5 (boy)	Fever, eye puffiness and redness, lip swelling, tongue redness, palm and sole rashes	RUQ pain, vomiting	WBC: 8,700/mm^3^	Pericholecystic fluid, gallbladder wall thickness	Minimal pericardial effusion: No evidence of coronary artery aneurysms	High-dose aspirin, IVIG, prednisolone: Recovery
				Hb: 11.3 g/dL			
				Platelet: 161,000/mm^3^			
				TB: 9.1, DB: 5.7 mg/dL, AST: 33, ALT: 27, ALP: 137 U/L			
Our case	0.25 (girl)	Fever	Diarrhea, RUQ pain, and guarding	WBC: 34,840/mm^3^	Marked distended gallbladder with internal bile sludge, gallbladder wall thickness, pericholecystic fluid	Perivascular brightness and generalized dilatation of all coronary arteries with a small aneurysm	High-dose aspirin, IVIG: Recovery
				Hb: 7.7 g/dL			
				Platelet: 929,000			
				ESR: 79 mm/h			
				CRP: 178.38 mg/dL			
				TB: 0.54, DB: 0.31 mg/dL, AST: 21, ALT: 10 U/L, albumin: 2.9 g/dL			
				Sodium: 134 mmol/L			

Our three-month-old girl presented with fever, diarrhea, and abdominal guarding. Initially, AAC was diagnosed as the cause of the infectious disease. Empirical antibiotics were administered for eight days without improvement. After conducting microbiological tests, including three blood samples, rectal cultures, and a real-time PCR test for HHV1-6, all returned negative results. Systemic non-infectious diseases, such as KD, were considered. Since this was a case of atypical KD in an infant, we were concerned about the 10-day delay in IVIG administration. Despite the absence of classic signs, additional blood tests and an echocardiogram suggested atypical KD. IVIG and high-dose aspirin were administered, which significantly improved the infant’s condition without any complications during the 1.5 years of follow-up.

Therefore, diagnosing patients who exhibit atypical symptoms at an uncommon age and lack classical signs of KD during initial presentation is challenging. This situation can lead to delays in diagnosis and management. Our case report emphasizes the importance of considering the possibility of KD in young infants with AAC, even in the absence of classic symptoms. To reduce the risk of KD-related complications, it is essential to include supplemental laboratory tests and perform echocardiography.

## Conclusions

KD is a common cause of systemic non-infectious AAC in previously healthy children, with an increased risk of coronary artery aneurysm. KD in infants may present atypically, with a lack of classical clinical manifestations. Misdiagnosed causes of AAC might lead to unnecessary surgical treatment rather than IVIG administration.
